# The Census of Great Britain

**Published:** 1882-07

**Authors:** 


### THE CENSUS OF GREAT BRITAIN.

On the night of April 4th the population of the United Kingdom of Great Britain and Ireland, so says the *Scientific American*, including the islands in British waters (the Isle of Man and the Channel Islands), together with the army and navy and merchant seamen abroad, was found to be 35,246,562, an increase of 4,147,236 as compared with the returns of the census of 1871. The females exceed the males by a little over 700,000. The percentage of population for England was 69·8; for Wales, 3·8; for Scotland, 10·6; for Ireland 14·6. The remainder, 1·2 per cent. was distributed between the Isle of Man (0.2), the Channel Islands (0·3), and the army, navy, and seamen abroad (0·7).

The density of population in England and Wales is 440 to the square mile. The greatest density is in the mining and manufacturing counties. Lancashire has over 1,700 to the square mile, and Middlesex (outside of London), 1,364. Six counties in England and one in Wales have over 500 to the square miles. London has 486,286 houses and a population of 4,814,571, having increased over half a million in the past ten years. The density of population in London is now 32,326 to the square mile.

Liverpool ranks next London in England, with a population over 550,000; Birmingham has over 400,000, Manchester and Leeds each exceed 300,000; Sheffield and Bristol have over 200,000 inhabitants each. Curiously the population of Manchester has fallen off 10,000 since the census of 1871.—*Canada Health Journal.*

